# Silk Vascular Grafts with Optimized Mechanical Properties for the Repair and Regeneration of Small Caliber Blood Vessels

**DOI:** 10.3390/ma15103735

**Published:** 2022-05-23

**Authors:** Elisa Valsecchi, Marco Biagiotti, Antonio Alessandrino, Dario Gastaldi, Pasquale Vena, Giuliano Freddi

**Affiliations:** 1 Silk Biomaterials Srl, 22074 Lomazzo, Italy; elisa@silkbiomaterials.com (E.V.); marco@silkbiomaterials.com (M.B.); antonio@silkbiomaterials.com (A.A.); 2 Laboratory of Biological Structures Mechanics-LaBS, Department of Chemistry Materials and Chemical Engineering Giulio Natta, Politecnico di Milano, 20133 Milano, Italy; dario.gastaldi@polimi.it (D.G.); pasquale.vena@polimi.it (P.V.)

**Keywords:** silk fibroin, small caliber vascular graft, morphological structure, mechanical performance, kink resistance, compliance

## Abstract

As the incidence of cardiovascular diseases has been growing in recent years, the need for small-diameter vascular grafts is increasing. Considering the limited success of synthetic grafts, vascular tissue engineering/repair/regeneration aim to find novel solutions. Silk fibroin (SF) has been widely investigated for the development of vascular grafts, due to its good biocompatibility, tailorable biodegradability, excellent mechanical properties, and minimal inflammatory reactions. In this study, a new generation of three-layered SF vascular scaffolds has been produced and optimized. Four designs of the SILKGraft vascular prosthesis have been developed with the aim of improving kink resistance and mechanical strength, without compromising the compliance with native vessels and the proven biocompatibility. A more compact arrangement of the textile layer allowed for the increase in the mechanical properties along the longitudinal and circumferential directions and the improvement of the compliance value, which approached that reported for the saphenous and umbilical veins. The higher braid density slightly affected the grafts’ morphology, increasing surface roughness, but the novel design mimicked the corrugation approach used for synthetic grafts, causing significant improvements in kink resistance.

## 1. Introduction

Cardiovascular diseases (CVDs) are the leading cause of mortality in the world, responsible for the deaths of over 17 million people a year [1]. According to the World Health Organization (WHO), the diseases of the cardiovascular system, in addition to always being severely debilitating, are responsible for almost 50% of deaths in Western countries, far exceeding the deaths from cancer, and the projections on global mortality estimate a rise up to 23.4 million deaths in 2030 [2,3]. An established treatment for coronary artery and peripheral vascular diseases is the implantation of vascular grafts to replace or bypass an occluded or damaged blood vessel. As a result, the demand for a suitable small-diameter (inner diameter < 6 mm) vascular graft is increasing globally [4].

Autologous grafts, such as saphenous vein or internal mammary artery, remain the gold standard in clinical practice [5], despite their limited availability, the surgical trauma due to their harvesting and their limited patency. Synthetic nondegradable materials, such as polytetrafluoroethylene (PTFE), expanded polytetrafluoroethylene (ePTFE) or polyethylene terephthalate (PET), can be considered as a replacement for large vessels due to their mechanical properties. However, these materials tend to fail for small-diameter grafts, owing to thrombogenicity complications and compliance mismatch between grafts and native vessels [6]. In addition, degradable synthetic materials, such as polyglycolic acid (PGA), poly-L-lactic acid (PLA), polylactide-coglycolide (PLGA) and polycaprolactone (PCL), have been investigated for vascular replacement, but owing to thrombus formation and their low mechanical properties, are not suitable for small-diameter vascular grafts [5]. Due to the limitations, a vascular substitute for small-diameter blood vessels remains an important issue to deal with vascular diseases. Considering the limited success of synthetic grafts, research has shifted towards biological solutions. Natural biopolymers, such as collagen, elastin and silk fibroin, offer better cytocompatibility and biocompatibility than synthetic grafts and can be considered better candidates for vascular grafts [7].

An ideal vascular graft must be biocompatible and hemocompatible, antithrombotic, compatible with vascular cells, biodegradable to allow vessel reconstruction without failure, elastic and flexible with adequate compliance with native vessels and mechanically robust to sustain blood pressure. Considering the mentioned requirements, vascular tissue engineering/repair aims to find a novel solution for vascular grafts. Recent research advancements brought the development of bioresorbable tissue-engineered vascular grafts (TEVG), which would be remodeled by host cells upon implantation, capable of promoting a native-like tissue formation comprised of tissue specific cells and extracellular matrix (ECM), using natural degradable polymers [8,9].

Among natural materials, *Bombyx mori* silk fibroin (SF), used for decades in its native form as suture [10] and more recently used in many biomedical applications [11], is one of the most preferred materials, due to its attractive properties. The interest in SF has substantially grown recently, due to its suitable mechanical properties, which make it an ideal solution in the regenerative medicine field [12]. The biological response to silk fibroin, if properly degummed and sterilized, is comparable to the most common biomaterials, since it enhances cells adhesion and, in vascular application, it does not induce thrombogenic effects [10]. Another essential feature is the capability to control the degradation rate of the scaffold, which should meet the tissue in-growth rate. Silk fibroin is subjected to enzymatic degradation by proteolytic enzymes (such as chymotrypsin, collagenase and actinase), which are responsible for hydrolysis of the peptide bond [13]. The in vivo biodegradation and reabsorption rate strongly depends on implantation site, physiological environment, patient’s state of health, physical or mechanical stress and SF morphology [12]. Material-related factors dictating the in vivo degradation of SF are mainly of structural (i.e., molecular weight, secondary structure, degree of crystallinity and orientation, hydrophobic/hydrophilic balance, accessibility of cleavage sites) and morphological (i.e., 2D/3D architecture, size, shape, density, porosity, and surface/mass ratio) origin [14,15]. In general, SF fibers lose most of their tensile strength in about one year in vivo [10]. The molecular structure of SF determines its exceptional mechanical properties. The high stability of SF fibers is owed to the hydrogen bonds, as well as to the hydrophobic nature and the high crystallinity of the protein [16]. The crystalline phase and the nanoscale conformation contribute to the determination of the mechanical properties of SF fibers, which are as follows: high mechanical tensile strength due to the presence of covalent bonds within the polypeptide chain and great flexibility due to the Van der Waals bonds joining β-sheet adjacent chains. The combination of these characteristics ensures greater toughness with respect to other biomaterials. Thanks to its excellent biocompatibility, controllable biodegradability, versatility of processing and excellent mechanical properties, silk fibroin is a suitable material for the development of small-caliber grafts and has been investigated in vascular tissue engineering for many years [17].

In the previous work, the chemical, morphological, physical, and mechanical properties of a novel multilayered SF tubular scaffold (called SILKGraft) were investigated [18]. The interaction with blood components was studied and the results of in vivo preliminary pilot tests on large animals were discussed. The process development led to a tubular device, characterized by easy handling during surgery and by a low biomechanical mismatch with the native artery; in addition to that, top level biomimicking performance with the surrounding living tissues can also be achieved [18]. In vitro studies with three main types of cells inhabiting the arterial wall, i.e., human coronary artery endothelial cells (HCAECs), human aortic smooth muscle cells (HAMSCs) and human aortic adventitial fibroblasts (HAAFs), showed a high degree of biocompatibility and a level of cell adhesion superior to polystyrene culture plates. Furthermore, blood hemocompatibility was corroborated by the lack of complement activation, hemolysis, and alteration of cell counts assays. The results of pilot animal studies indicated sheep as the model of choice to carry out the in vivo tests before the clinical trials in humans and that the device is easy to use regarding surgical stitching and handling [18].

Despite these promising results, the graft showed some mechanical limitations in terms of low resistance to kinking, which might reduce the blood flow when the graft bends. In this study, the optimization of the mechanical properties of the graft was addressed in order to increase kink resistance. To address this issue, two modifications were implemented during the production of the tubular scaffold, while keeping the three-layered design of the wall unchanged, in order to maintain its previously proved biomimicking attitude and performance. Combining the changes, three new designs, in addition to the standard SILKGraft design previously reported [18], were obtained and characterized from the chemical, morphological, physical, and mechanical point of view. The improvement achieved against kinking was assessed through the bendability test, which is aimed at evaluating the ability of the graft to maintain its shape (internal lumen) when implanted in joints subjected to bending; furthermore, the compliance was evaluated and discussed.

## 2. Materials and Methods

### 2.1. Production of the SILKGraft Vascular Prosthesis

The SILKGraft vascular prosthesis was manufactured as previously reported [18]. Briefly, to make the wall of the device, two electrospun (ES) layers were assembled onto the inner and outer faces of a tubular textile (TEX) braid, according to a patented process [19]. The coupling of the TEX layer with the two ES layers was made during electrospinning by means of a welding medium consisting of an 80% *v*/*v* aqueous solution of the ionic liquid 1-ethyl-3-methylimidazolium acetate (EMIMAc; #51053, Sigma-Aldrich, Milano, Italy). The same welding medium (EMIMAc/H_2_O) was used to couple the first and second ES layers to the inner and outer surface of the TEX layer, respectively. After electrospinning, the three-layered ES/TEX/ES tubular structure was consolidated by immersion in 80% *v*/*v* ethanol/water for 30 min at room temperature, followed by overnight washing with distilled water and drying. The vascular prosthesis produced according to this standard method was identified with the acronym SILKGraft-1 (Table 1).

Three designs are proposed here as variation with respect to the standard design SILKGraft-1. These variants of the basic SILKGraft-1 structure were produced by varying the way the tubular TEX layer is assembled onto the electrospinning mandrel and the composition of the welding medium. In particular, the design SILKGraft-2 was obtained using a higher density arrangement of the TEX braid when mounted onto the mandrel before electrospinning, keeping all the other manufacturing parameters the same as those of the SILKGraft-1 variant. The higher density arrangement of the TEX braid resulted in an increase in the number of crossing points per unit length (cm), where the yarns interlace each other. SILKGraft-1 Plus and SILKGraft-2 Plus, with standard and higher density braid arrangements, respectively, were obtained by using two different welding media, which were as follows: (i) EMIMAc/H_2_O to couple the first ES layer to the inner TEX surface; (ii) a solution of 15% *w*/*w* silk fibroin in EMIMAc (SF/EMIMAc) to couple the second ES layer to the outer TEX surface. Table 1 shows schematically the manufacturing changes in each design.

### 2.2. Morphological Characterization

#### 2.2.1. Surface Morphology

The surface morphology was analyzed with a scanning electron microscope (SEM; Zeiss EVO MA10, Oberkochen, Germany) on Au/Pd sputter-coated samples (Desk IV, Denton Vacuum, LLC, Moorestown, NJ, USA), at 10 kV acceleration voltage, 100 mA beam current, and 15 mm working distance. The tubular device (length = 100 mm) was cut longitudinally, flattened, and three specimens of 10 × 10 mm were sampled along its length and mounted on aluminum stubs with the surface of the inner electrospun layer exposed. Three tubular devices were analyzed for each variant.

#### 2.2.2. Wall Thickness

The wall thickness was measured according to the ISO 7198:2016 standard method. The tubular device (length = 100 mm) was cut longitudinally, flattened, and measured in seven different positions along its length with a thickness tester MarCator 1075R (Mahr, Göttingen, Germany), equipped with a constant load thickness gauge of 0.3 cm^2^ foot area that exerted a pressure of 1 kPa. Three tubular devices were analyzed for each variant.

#### 2.2.3. Relaxed Internal Diameter

For the measurement of the relaxed internal diameter, a cross-section of the tubular devices was mounted on aluminum stubs and prepared for SEM analysis. A low magnification image of the cross-section was acquired, and the internal diameter was determined using the SEM software measuring tools. Three cross-sections taken at different positions along the length of each device were measured. Three tubular devices were analyzed for each variant.

#### 2.2.4. Surface Roughness

The surface roughness of the inner ES layer was determined in axial and circumferential directions according to the DIN 4768 standard method. The tubular device (length = 100 mm) was cut axially, flattened, and an area of 25 mm^2^ of the inner surface was analyzed, with a scanning length of 9 mm and a scanning density of 150 points per mm. The centerline average roughness height (R_a_, arithmetic average of the absolute values of the profile heights over the evaluation length), root mean square roughness height (R_q_, root mean square average of the profile heights over the evaluation length), and maximum peak to through height (R_t_, average value of the absolute values of the heights of five highest-profile peaks and the depths of five deepest alleys within the evaluation length) were calculated. The analysis was repeated in five different positions for each device. Six tubular devices were analyzed for each design, SILKGraft-1 and SILKGraft-2.

### 2.3. Spectroscopic Characterization

The attenuated total reflectance Fourier transform infrared spectroscopy (ATR-FTIR) analysis was performed with an ALPHA FTIR spectrometer equipped with an ATR platinum diamond accessory, at a resolution of 4 cm^−1^, in the infrared range of 4000–400 cm^−1^. The tubular specimen was cut longitudinally and the surface of the inner electrospun layer was pressed against the ATR crystal. Two spectra were recorded in different positions for each device and averaged. The spectra were corrected with a linear baseline and normalized to the CH_2_ bending peak at about 1445 cm^−1^. This peak was selected because it is not sensitive to SF molecular conformation. The crystallinity index was calculated by ratioing the intensity of the amide III bands at 1262 cm^−1^ and 1227 cm^−1^ (CI = A_1262_/A_1227_) [20]. Three tubular devices were analyzed for each variant.

### 2.4. Thermal Characterization

Differential scanning calorimetry (DSC) analyses were performed with a DSC 3500 Sirius (Netzsch, Selb, Germany). The samples (3–5 mg) were sealed in aluminum pans and subjected to a heating cycle from 50 to 400 °C, at a heating rate of 10 °C/min, under N_2_ atmosphere (flow rate: 20 mL/min). Each device was analyzed in duplicate. Three tubular devices were analyzed for each variant. The following parameters were determined from the DSC curves: peak temperature of the endotherms associated with melting/degradation of the ES (T_ES_) and TEX (T_TEX_) layers; cumulative enthalpy (ΔH_ES/TEX_) of the ES and TEX endotherms; ES:TEX weight ratio, calculated from the relative intensity of the respective DSC endotherms, using the ΔH values of −402 J/g and −307 J/g for the pure TEX and ES layers, respectively, to normalize the contribution of the two materials [18].

### 2.5. Mechanical Characterization

#### 2.5.1. Circumferential Tensile Test

The purpose of this test is to determine the circumferential tensile strength of the sample prosthesis in its tubular form when placed onto two rounded pins and stretched at a uniform rate, until the break point is reached. The tests were performed according to ISO 7198:2016 (Annex A, Subclause A.5.2.4.), using an All-Electric Dynamic Test Instrument ElectroPuls E3000 (Instron, Norwood, MA, USA), equipped with a load cell of 250 N, a thermostatic bath (BioPuls, Instron, Norwood, MA, USA), and appropriate custom-made grips (Figure S1A; Supplementary Materials). The samples (length = 10 mm) were cut carefully, mounted on the grips, conditioned in water at 37 °C for 5 min, and tested while submerged at a crosshead speed of 50 mm/min. Each device was analyzed in triplicate. The load–displacement curve was obtained and the maximum load (N) of three tubular devices for each design was evaluated. The elastic modulus (MPa) was then calculated by the following equation

(1) $$E=\frac{\sigma }{\varepsilon }$$

where σ (MPa) indicated the engineering stress and ε (mm/mm) was the correspondingly strain, determined as the ratio between Δl and $l_{0}$. The engineering stress was calculated as follows:

(2) $$\sigma =\frac{F}{A}$$

where F indicated the load (N) in circumferential direction and A (mm^2^) corresponded to the cross-section area, determined as A=2s×l, where s and l were the wall thickness and length of the sample, respectively.

#### 2.5.2. Uniaxial Tensile Test

The purpose of this test is to determine the longitudinal tensile strength of the sample prosthesis in its tubular form when loaded longitudinally along their centerline. The samples were stretched at a uniform rate, until the break point is reached. The test was performed according to ISO 7198:2016 (Annex A, Subclause A.5.2.3.), using a Single Column Load Frame Test Instrument E3345 (Instron), equipped with a load cell of 500 N. The samples (length = 100 mm) were conditioned in water at 37 °C overnight, then were mounted on the grips and tested at a crosshead speed of 50 mm/min with a preload of 0.05 N (Figure S1B; Supplementary Materials). The gauge length was 50 mm. The load–displacement curve was obtained. The maximum load (N) and the displacement (mm) values of the three tubular devices for each design were evaluated.

#### 2.5.3. Pressurized Burst Strength

Burst pressure testing is a direct approach to determine the maximum internal pressure a tubular device, such as a vascular graft, withstands before failure. The test was carried out according to ISO 7198:2016 (Annex A, Subclause A.5.2.2.). The sample (length = 100 mm) was placed in an adapter that fits the internal diameter of the graft. At one end, a tube connected the graft to a manometer and a syringe, while a pressure sensor was located at the other end, where a 3-way-luer lock was turned to close the connection to a reservoir. Before testing, the whole system was rinsed with saline solution. Then, the sample and the perfusor lines were filled with porcine heparinized blood, while the syringe was filled with saline solution and pressed to provide a pressure ramp. The pressure in the graft was measured continuously by the located sensor upstream the sample and recorded by the data acquisition system (DAQ-software), until the maximum pressure was reached and the sample burst. The burst pressure was identified as the pressure level at which damage in the outer ES layer occurs. The burst pressure resistance (mmHg) and the mean diameter (mm) of bursting damage were evaluated on three tubular devices for each selected design, i.e., SILKGraft-1, which represents the standard reference device, and SILKGraft-2 Plus, which incorporates both process changes.

#### 2.5.4. Kink Resistance

The test was performed according to ISO 7198:2016 (Annex A, Subclause A.5.8.). The sample (length = 100 mm) was placed in an adapter that fits the internal diameter of the graft, filled with porcine heparinized blood, and maintained at a constant pressure of 100 mmHg. To provide a hydrostatic pressure of 100 mmHg, a blood bag was used at a fixed height and connected to the pressure sensor through a tube. The sample was finally bent in radius templates with different internal diameters decreasing from 54 mm to 18 mm, with decrements of 3 mm. The diameter of the template that first causes graft kinking was recorded and the test was concluded. Three tubular devices were analyzed for each design, i.e., SILKGraft-1, which represents the standard reference device, and SILKGraft-2 Plus, which incorporates both process changes.

#### 2.5.5. Compliance

The static radial compliance was measured by means of pressure-driven inflation tests, recording the change in volume of the tubular device. The sample (length = 120 mm) was filled with bovine blood and mounted on two rigid connectors, one for each end. The upstream connector was attached to the rigid tube coming from a syringe, while at the other end the connector was attached via luer lock to a pressure transducer. A syringe pump provided the flow through the sample and the pressure values were recorded with an acquisition frequency of 200 Hz. Before testing, the samples were rinsed with saline solution at room temperature. The test was conducted using a constant flow rate of 0.27 mL/sec, which allowed for the change in the internal pressure of the sample from 80 mmHg to 120 mmHg in about 0.5 s, simulating a cardiac cycle. The pressure values, recorded with an acquisition system, were evaluated with a frequency of 200 Hz. The test was concluded when the blood came out from the sample or when an internal pressure of 250 mmHg was exceeded. Compliance (C) was calculated using the following equation:

(3) $$C=\frac{\Delta V_{\left(80-120\right)\mathrm{mmHg}}}{\Delta P}$$

where $\Delta V_{\left(80-120\right)\mathrm{mmHg}}$ is the volume variation corresponding to a pressure variation between 80 and 120 mmHg and ΔP is the pressure variation between 80 and 120 mmHg. The volume measurement is obtained indirectly, multiplying the constant flow rate by the time needed to increase the internal sample pressure from 80 to 120 mmHg. Three tubular devices were analyzed for each design, i.e., SILKGraft-1, which represents the standard reference device, and SILKGraft-2 Plus, which incorporates both process changes.

### 2.6. Statistical Analysis

Data were expressed as mean values ± SE. The comparison between each design started with the analysis of population normality and their level of statistical significance was assessed by means of F-Test and *t*-Test. A value of *p* < 0.05 was taken as significant.

## 3. Results

### 3.1. Geometrical and Morphological Characterization

**Figure 1:**
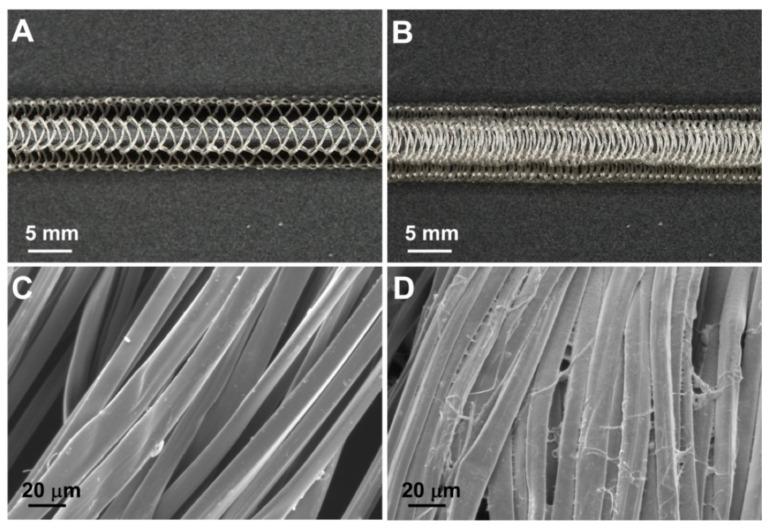
Design changes of the manufacturing process. Images (**A**,**B**) were obtained with a digital microscope (Omni 3 Ash, Naas, Ireland); images (**C**,**D**) were obtained with SEM (Zeiss EVO MA10). (**A**) Extended arrangement of the braid (TEX layer of the SILKGraft wall) on the electrospinning mandrel. Crossing points per unit length: 6/cm. (**B**) Compact arrangement of the braid on the electrospinning mandrel. Crossing points per unit length: 14/cm. (**C**) Detail of the yarn forming the TEX layer after treatment with the EMIMAc/H_2_O welding agent. After treatment with the hydroalcoholic solution (80 v% ethanol in water), the welding agent is completely removed and does not leave any residuals on the surface of the fibers, which remain separated from each other. (**D**) Detail of the yarn forming the TEX layer after treatment with the SF/EMIMAc welding agent. After treatment with the hydroalcoholic solution (80 v% ethanol in water), the welding agent leave a thin SF film on the surface of the yarns that glues together the constituent fibers.

Figure 1 shows the design changes introduced in the manufacturing process of the SILKGraft vascular prosthesis. Two ways of assembling the TEX braid onto the electrospinning mandrel were combined with the use of two different welding media. The extended arrangement of the braid (Figure 1A) was used to produce the two SILKGraft-1 designs (SILKGraft-1 and SILKGraft-1 Plus). In this configuration, the voids between the braid meshes were clearly observable. The compact arrangement of the braid (Figure 1B) was adopted to produce the SILKGraft-2 designs (SILKGraft-2 and SILKGraft-2 Plus). As shown in the picture, the voids between the meshes were much smaller than those shown by SILKGraft-1. The compacted configuration in SILKGraft-2 and SILKGraft-2 Plus resulted in an increase in the number of crossing points per unit length (cm), where the yarns interlace each other. SILKGraft-1 designs showed 6 crossing points per unit length (cm), while SILKGraft-2 designs showed 14 crossing points per unit length (cm). In addition to this, for each SILKGraft group, the variant “Plus” was manufactured by using a different welding medium to couple the second ES layer to the outer TEX surface. The welding medium EMIMAc/H_2_O was used in SILKGraft-1 and SILKGraft-2; while the medium SF/EMIMAc was used for the “Plus” designs. At the end of the manufacturing process, the former welding medium was completely removed by the extraction/washing steps, leaving the fibers clean and smooth (Figure 1C), while the latter welding medium left a thin SF film on the surface of the yarn (Figure 1D). This SF film glued together the constituent microfibers, leading to the consolidation of the network and less freedom of movement of the microfibers/yarns, with respect to each other.

The four SILKGraft designs developed in this study were characterized to evaluate the impact of the different manufacturing techniques, i.e., the textile braid density and the use of different welding agents, on their geometrical and morphological properties (Table 2). Comparing the four designs, the use of different welding agents did not significantly influence the wall thickness within the same SILKGraft group (1 or 2). Each design of the SILKGraft-2 group displayed a statistically significant higher wall thickness than each design of the SILKGraft-1 group. These results indicate that the wall thickness did not depend on the welding agent used, i.e., EMIMAc/H_2_O or SF/EMIMAc, but was mainly determined by the arrangement of the braid; the more compact the braid, the thicker the wall. The same comments apply to the internal diameter, which significantly increased at higher braid densities.

**Figure 2:**
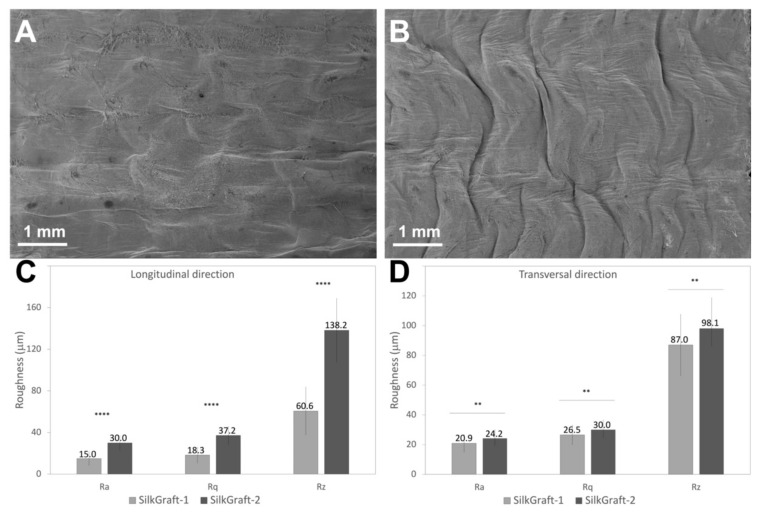
(**A**) Typical SEM surface morphology of the inner ES layer of the SILKGraft-1/1 Plus designs. (**B**) Typical SEM surface morphology of the inner ES layer of the SILKGraft-2/2 Plus designs. (**C**,**D**) are plots of the average values of R_a_, R_q_, and R_z_ measured in the longitudinal and transversal directions, respectively. Statistical significance: *p* < 0.0001 (****); *p* < 0.01 (**). The source data used for the graphs are reported in Tables S3 and S4 for the longitudinal and transversal direction, respectively (Supplementary Materials).

The surface morphology of the inner ES layer of the devices was evaluated by SEM analysis. The SILKGraft devices with lower mesh densities (1 and 1 Plus) showed a smooth surface (Figure 2A). The texture of the underlying TEX layer could be perceived through the contact points with the overlying ES layer. The increase in mesh density (2 and 2 Plus) resulted in a completely different surface morphology, with the presence of ridges with peaks and valleys running almost axially (Figure 2B). The change in welding media did not affect the surface morphology of the lumen of the device.

The roughness of the inner surface of the tubular devices was quantitatively evaluated on the SILKGraft-1 and SILKGraft-2 designs as representative models of the low and high braid density samples, respectively. The complete set of surface roughness data obtained from the measurements taken in the axial and circumferential directions is reported in Tables S1 and S2, respectively (Supplementary Materials). A comparison of the average values of R_a_, R_q_, and R_z_ obtained in the axial and circumferential directions is shown in Figure 2C,D. Statistically significant higher values of the three roughness parameters were obtained for SILKGraft-2 in the axial direction, as a consequence of the presence of ridges running perpendicular to the measurement direction. On the other hand, the roughness values measured in the circumferential direction were closer, although still statistically different.

### 3.2. Thermal and Spectroscopic Characterization

**Figure 3:**
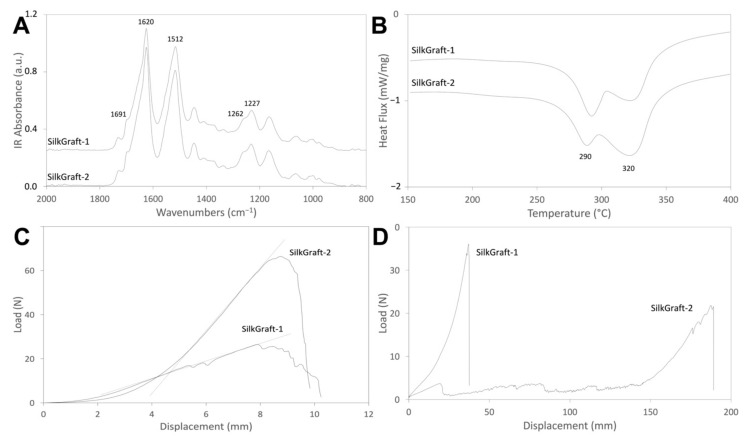
(**A**) ATR-FTIR spectra of SILKGraft designs 1 and 2. (**B**) DSC thermograms of SILKGraft designs 1 and 2. (**C**) Circumferential tensile curves of SILKGraft designs 1 and 2. (**D**) Longitudinal tensile curves of SILKGraft designs 1 and 2.

The structural properties of the inner ES layers that compose the four SILKGraft designs were characterized by ATR-FTIR. Two representative spectra for the SILKGraft 1 and SILKGraft 2 groups are shown in Figure 3A. No differences were observed in terms of band position and intensity. All the spectra showed the typical profile of β-sheet crystalline silk fibroin materials, with amide I at 1620 cm^−1^ (shoulder at 1691 cm^−1^), amide II at 1512 cm^−1^, and amide III at 1227 cm^−1^ and 1262 cm^−1^ [18]. The crystallinity index of the ES nanofibers, expressed as an intensity ratio between the two amide III components (CI = I_1227_/I_1262_), are reported in Table 3. The values fall in the range characteristics of native SF microfibers (CI ≅ 0.60) [21] and regenerated SF nanofibers (CI ≅ 0.59) [22]. Interestingly, a slight increase in the crystallinity index can be observed for the SILKGraft designs of group 2. No significant differences were recorded between each design and its Plus version.

Figure 3B shows representative thermograms of the SILKGraft devices belonging to group 1 and 2. The DSC curves displayed two broad endothermic transitions [18], one peaking at about 320 °C, attributed to the melting/degradation of the native SF fibers comprising the TEX layer [21], and the other falling at a lower temperature, with a peak at about 290 °C, attributed to the thermal degradation of the regenerated ES nanofibers [23]. The values of the ES and TEX peak temperatures and of enthalpy did not show statistically significant differences between the four SILKGraft designs (Table 3). The main difference between the two SILKGraft groups was the relative intensity of the TEX endotherm (Figure 3B), which increased for the samples of group 2 made with the braid in the compact configuration. As a result, the weight percentage of the ES layers comprising the wall of the devices decreased in a statistically significant mode going from group 1 to group 2 (Table 3), because of the presence of a relatively higher amount of braid per unit length.

### 3.3. Mechanical Characterization

#### 3.3.1. Circumferential Tensile Test

Two representative examples of the typical curves of SILKGraft groups 1 and 2 are shown in Figure 3C, while the data are listed in Table 4. The characteristic load/displacement profile comprised an initial flat region with substantial increase in deformation for the low values of the load, followed by an almost linear load/displacement ramp with a different slope between the two groups of samples and, finally, a breaking point. The values of the elastic modulus and maximum load were significantly higher for the samples with high braid density (SILKGraft-2 group). Within the same SILKGraft group, the change in welding agent had no impact on the circumferential tensile properties of the devices.

#### 3.3.2. Uniaxial Tensile Test

Two representative examples of the typical curves of SILKGraft groups 1 and 2 are shown in Figure 3D, while the data are listed in Table 4. Each design of the SILKGraft-2 group displayed a statistically different normalized maximum load and displacement at the maximum load, when compared to each design of the SILKGraft-1 group. The behavior of the samples was strongly influenced by the braid density. The SILKGraft-1 group samples presented the TEX layer already well extended in the direction of load application. After a short initial stage of fiber recruitment, the linear load/displacement ramp started leading to final rupture. The final value of maximum load included the contributions of both the ES and TEX components. On the other hand, the SILKGraft-2 group samples displayed a deformation phase at relatively low load values, which was substantially larger than that exhibited by SILKGraft-1. Numerous small peaks, which marked the progressive detachment of the ES layer from the TEX component and the breakage of the ES nanofibers, were observed. When the extension of the TEX layer was completed, the load increased until rupture. Breakage occurred at lower load values, due to the loss of the ES layers’ contribution that had been dispersed between the previous small rupture peaks (Figure 3D).

#### 3.3.3. Pressurized Burst Strength

The representative samples of the SILKGraft group 1 and group 2 were analyzed, and the results are reported in Table 5. The average value of burst pressure was higher for the sample SILKGraft 2-Plus, but not statistically different from the SILKGraft-1 sample. In addition, the measurement of the mean diameters of the bursting damages did not return statistically different results.

#### 3.3.4. Kink Resistance

**Figure 4:**
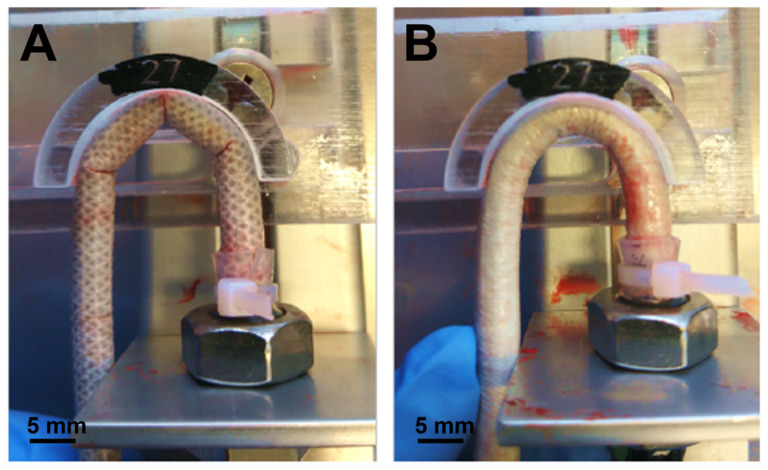
Kink resistance test. Images of the SILKGraft-1 (**A**) and SILKGraft-2 Plus (**B**) devices under kink resistance testing with the 27 mm diameter adapter. The SILKGraft-1 device shows three evident kinking points in the middle and in the initial and final positions of the adapter.

The values of the bending diameter required to produce a kink were determined on the representative samples of SILKGraft groups 1 and 2 and the results are listed in Table 5. The kinking effect started appearing at smaller bending diameters (i.e., higher curvature) for the samples of the group 2, indicating that the higher braid density increased the ability of the device to withstand bending stresses. When perfused with porcine heparinized blood at 100 mmHg pressure and placed in the testing device with the same bending diameter, the SILKGraft-1 sample kinked in three different points in each bending configuration tested, while the SILKGraft-2 sample showed an excellent kink resistance without kinking points, until a significantly lower value of bending diameter (Figure 4). These data demonstrate that a higher mesh density of the TEX layer may improve the kink resistance when the graft is bent; the values of the bending diameter required to produce a kink were smaller for SILKGraft-2 Plus than SILKGraft-1 and the difference was statistically significant.

#### 3.3.5. Compliance

Table 5 lists the values of compliance of the samples of SILKGraft groups 1 and 2. The compliance was higher for the sample of the SILKGraft-2 group, and the difference was statistically significant. The axially extended TEX layer probably opposed some resistance to radial deformation within the 80–120 mmHg pressure range.

## 4. Discussion

The translation of a medical device from the research scale to clinical settings requires continuous efforts to adjust and improve its characteristics, not only to comply with the stringent regulatory requirements but also to meet the clinical challenges targeted by the application. In summary, a device must function as intended by its use.

The SILKGraft vascular prosthesis is a tubular device whose wall consists of two electrospun (ES) layers coupled to an intermediate textile (TEX) layer [18]. A key aspect of the manufacturing process is the capability to obtain a multilayer structure, in which the different layers are intimately integrated at the structural and functional level and respond as a single body to mechanical stresses, without showing mutual slipping or separation, a prerequisite to avoid biomechanical mismatch when implanted in vivo. *Bombyx mori* silk fibroin (SF) was chosen as the starting material to manufacture both the ES and the TEX layers. SF is a biopolymer unanimously recognized as biocompatible that has been proposed for a wide range of medical applications for the regeneration/repair of both soft and hard tissues [11]. Not surprisingly, the SILKGraft vascular prosthesis demonstrated good biocompatibility and hemocompatibility in vitro. Moreover, in vivo short term (1 month) pilot trials on large animal models (minipig and sheep) gave encouraging results and allowed for the planning of long-term in vivo studies that are currently in progress.

The SILKGraft device produced with the basic manufacturing process [18], here identified with the acronym SILKGraft-1, has demonstrated good mechanical properties in terms of circumferential tensile strength and pressurized burst strength. However, it has shown some limitations regarding its resistance to bending stresses, a phenomenon that can lead to cause either a discontinuity on the inner radius or a reduction in lumen diameter during the bending deformation. This effect, also known as kinking, can cause severe turbulence of the blood flow or even hinder the proper passage of blood, leading to the failure of the implant [24]. The SILKGraft vascular prosthesis is currently produced with a length of 10 cm. When a prosthesis of this length is implanted, it remains in a straight position and is unlikely to undergo bending stresses. However, the vascular prostheses commercially available and used in clinics are longer and it is highly probable that some parts fall in correspondence to the joints, and are, therefore, subject to bending. For this reason, the demonstration of good resistance to kinking is a fundamental requirement for a vascular prosthesis.

To address the issue of improving the kink resistance of the SILKGraft vascular prosthesis, an innovation in the graft design was proposed, while keeping the manufacturing process unchanged as much as possible, which has already proved to be robust and reproducible. In particular, the three-layered design of the wall structure, which grants the biomimicking attitude of the ES layers and the mechanical support provided by the intermediate TEX layer, was maintained. It was, therefore, decided that one would intervene on two construction parameters that could have been easily managed within the same production process, i.e., the arrangement of the braid and the composition of the welding agent used to couple the braid to the ES layers. This choice was based on the assumption that a compact arrangement of the braid could allow it to extend more easily along the side where the bending tension is applied, thus, keeping the shape of the tubular structure unchanged. Moreover, the consolidation of the yarn obtained with the deposition of a silk fibroin film on the fibers could provide greater resistance to transversal deformation, thus, avoiding the collapse of the tube and the reduction in the lumen diameter by the kinking of the region where the bending compression acts.

The implementation of the above changes allowed the production of four designs of the vascular device that were characterized from a morphological and chemical-physical point of view in order to evaluate the effects of the changes in the devices’ properties. While the change in welding agent, i.e., the use of the SF/EMIMAc solution, which left a thin SF film on the surface of the fibers of the TEX layer, had only negligible effects, the increase in braid density had a strong impact on the wall thickness and internal diameter (Table 2). While the increase in wall thickness may be a direct result of the more compact braid arrangement, the increase in cross-sectional diameter also reflected the contribution of other factors. The three-layered tubular devices were built on 5 mm diameter mandrels. After removal from the mandrel, the tubular structure was consolidated by immersion in aqueous ethanol to crystallize the ES layers by inducing a random coil to β-sheet conformational transition [25]. The molecular rearrangement occurring in the bulk of the ES layers caused dimensional changes in all directions. With reference to the transversal size of the tubular structure, shrinking resulted in the decrease in the nominal diameter from 5 mm to about 4.7 mm with the braid in the extended, less compact state (SILKGraft-1 group). On the other hand, in its more compact configuration, such as in the SILKGraft-2 group, the braid offered a higher resistance to shrinkage. This behavior, in addition to the higher starting diameter due to the different arrangement of the braid, brought a final diameter of the device that was practically identical to the nominal one.

The change in braid density significantly affected the morphology of the inner surface of the device, causing an overall increase in surface roughness, especially in the longitudinal direction (Figure 2). The closing of the voids between the meshes and the approaching of the braid nodes caused by the more compact arrangement of the braid allowed a greater number of contact points per surface unit to be formed between the TEX and the ES layers. The shrinkage of the ES layers during the hydroalcoholic treatment, which resulted from the rearrangements occurring at the molecular level [26], further stabilized the wrinkled structure of the inner surface, as demonstrated by the SEM micrographs and the roughness results. The aortic endothelium is rough at the submicron scale, with ridges and grooves that are generally aligned in the blood flow direction [27]. These topographical features may offer increased cellular attachment as well as alignment in the direction of blood flow, providing a better hemocompatibility. A future evaluation to verify if the increased surface roughness affected graft hemocompatibility will be carried out.

The chemical-physical properties of the devices were not affected by the manufacturing changes. Both the thermal and spectroscopic data (Figure 3, Table 3) accounted for a full crystallization of the regenerated component of the device, confirming that the treatment with the hydroalcoholic solution allowed the completion of the conformational transition from a prevalently random coil to a β-sheet crystalline structure of the ES layer, as also evidenced by the values of CI calculated from the FTIR spectra [28]. These physical and structural characteristics of the materials comprising the device are of chief importance because they may impact the biological interaction with the surrounding tissues [29] and the rate and extent of degradation upon implantation (the device is entirely made from a biodegradable natural protein polymer) [13]. The device components have been designed to degrade in the medium (ES layers) to long term (TEX layer) in order to allow mechanical support during the first stages after implantation, and then to gradually transfer the load-bearing capacity to the new vascular tissue infiltrated within the slowly degrading wall of the prosthesis. Both the FTIR and DSC results confirmed the structural integrity of the devices’ components, thus, excluding any impact of the manufacturing changes on the expected biological performance upon implantation, due to the physical and structural characteristics evaluated.

The mechanical characteristics of the SILKGraft designs were strongly influenced by the higher longitudinal density of the braid (Table 4). The increase in braid density per unit length has led to a doubling of the load and modulus values measured in the circumferential direction, confirming that the TEX layer is the load-bearing component of the device. As discussed in the previous work, the contribution of ES layers is negligible [18]. The maximum load values in the circumferential direction of the SILKGraft-2 groups were significantly higher than those of the autologous vessels used as gold standard, such as the saphenous vein [30]. The braid density also influenced the mechanical response in the longitudinal direction, as the elongation at break was more than four times larger for the SILKGraft devices of the group 2 due to the extendibility of TEX layer. Instead, the maximum load of the SILKGraft designs in the longitudinal direction was comparable to those of natural vessels [30]. The difference between the maximum load showed along the circumferential and longitudinal orientations reflected the anisotropic behavior of the SILKGraft designs. This direction dependance could be observed also in the native blood vessels, owed to their anisotropic structures.

The burst pressure was not statistically different between the designs of group 1 and 2. The average value of about 1000 mmHg was lower than that previously reported (2308 mmHg) [18], due to different experimental approaches. The previous test was made with a balloon placed inside the tubular graft, which caused a pressure increase on a large portion of the inner surface. While in this case, the pressure ramp was provided by perfusing and pressing porcine heparinized blood directly into the ES layer of the graft and the pressure increase was sustained by a much smaller portion of the inner surface. The pressure drop occurred as soon as the ES layer broke, while the TEX layer was still intact. Nevertheless, the value recorded in this study was still higher than that reported for vascular grafts made only of electrospun SF fibers with a similar diameter [23].

The mechanical response of small caliber vascular grafts to bending stresses depends on many factors, including the intrinsic properties of the constituent polymer, the manufacturing process, and the design of the wall of the tube [31]. Regenerated SF materials, such as films and sponges, may be quite brittle or stiff, thus, being unsuitable for manufacturing kink resistant vascular grafts. However, the use of the electrospinning technology may pose less restrictions to the development of a tubular structure with good elasticity to reduce the impact of kink formation [32]. Further improvement can be achieved with the adoption of multilayered wall architectures, as in the case of the SILKGraft design, which aimed at combining the good elasticity in the wet state of electrospun layers, with the strength and ductility of an intermediate reinforcing braided layer [18]. However, the kink resistance may remain a limiting performance characteristic (Table 5, Figure 4). Kink resistance is a fundamental property for a vascular graft, as the kinking phenomenon can cause partial or total occlusion of the device lumen and interruption of the flow, with very serious consequences for its functionality and, when implanted, patient’s life. A recent trend to overcome the bottleneck of thepoor kinking behavior of polymeric tubular structures targeting vascular tissue regeneration is the adoption of manufacturing solutions resulting in an effective reinforcement of the tube in the radial direction, such as the insertion of spiral structures deposited by 3D printing [24] or the corrugation of the wall of the tube [33]. Significant improvements of the kink resistance were reported with both approaches. In the case of the SILKGraft designs developed in this study, the grafts with denser braid arrangement mimicked the corrugation approach. The higher extensibility of the intermediate TEX layer allowed by the braid density (Table 4, Figure 3D) could follow the deformation of the ES layers on the external tension side of the tube, while the density of the braid in the internal compression side prevented the ES layers from yielding and forming kinks. As a result, the kink diameter of the SILKGraft-2/2 Plus variant has been significantly improved, reducing to half that of the SILKGraft-1/1 Plus design.

Finally, a significant improvement of the compliance was obtained with the denser braid arrangement (Table 5). Compliance mismatch between the native artery and the graft is unanimously considered the leading cause of failure for small caliber vascular grafts and many efforts are made to develop a vascular prosthesis with compliance values as close as possible to those of natural blood vessels [34]. Previously developed electrospun SF grafts [23] have shown compliance values superior that those of the currently available synthetic grafts made of PET or ePETF [34]. The three-layered SILKGraft-1 variant with the braid in the extended arrangement displayed a compliance value of 3.23 mL/mmHg × 10^−3^. On the other hand, the compact braid arrangement, which probably gave the tubular structure more freedom to accommodate in the radial direction the deformation produced by the blood pressure, resulted in the value of 3.75 mL/mmHg × 10^−3^, closer to the compliance reported for the saphenous and umbilical veins, which nowadays represent the gold standard for the replacement of small caliber arteries [35].

## 5. Conclusions

Currently, the need for small-diameter vascular graft is increasing globally. For the past two decades, silk has been investigated as a potential candidate to create tissue engineered grafts. With regard to the biomedical application of this natural biopolymer, a vascular prosthesis (SILKGraft) consisting of a tubular device obtained with pure silk fibroin was developed. SILKGraft was made of two electrospun (ES) layers and an intermediate TEX layer and demonstrated good biocompatibility, hemocompatibility and good mechanical properties. Despite the encouraging results obtained, SILKGraft needed some improvements, regarding its kink resistance. To enhance this mechanical property, the SILKGraft structure consisting of three SF layers was maintained unaltered and two parameters were changed, including the greater density of the braid arrangement and the composition of the welding agent used to couple the braid to the ES layers. The SILKGraft designs were investigated and showed enhanced mechanical properties in terms of circumferential and longitudinal tensile strength, strongly influenced by the higher braid density. Another extremely important mechanical property to take into account is the compliance. The investigation made on the SILKGraft designs clearly showed that braid arrangement increased the ability of the tubular structure to allow deformation in the radial direction produced by the blood pressure. Finally, a significant improvement in kink resistance was obtained. The braid arrangement provided a higher extensibility and prevented graft occlusions. Therefore, a higher braid density allowed an excellent improvement in the mechanical properties, especially in the scaffold bendability.

## Figures and Tables

**Table 1:** Acronyms of the design variants of SILKGraft.

		**Braid Density ^2^**	
		**(a) Low**	**(b) High**
**Welding media ^1^**	(c) EMIMAc/H_2_O	“SILKGraft-1” (a/c)	“SILKGraft-2” (b/c)
	(d) SF/EMIMAc	“SILKGraft-1 Plus” (a/d)	“SILKGraft-2 Plus” (b/d)

^1^ EMIMAc/H_2_O: aqueous solution of EMIMAc in water 80/20% *v*/*v*. SF/EMIMAc: 15% *w*/*w* silk fibroin in EMIMAc. ^2^ Crossing points per unit length: low = 6/cm; high = 14/cm.

**Table 2:** Geometrical and morphological characteristics.

SILKGraft Design	Wall Thickness (mm)	Internal Diameter (mm)
SILKGraft-1	0.56 ± 0.03	4.65 ± 0.06
SILKGraft-1 Plus	0.54 ± 0.05	4.66 ± 0.15
SILKGraft-2	0.79 ± 0.11	4.98 ± 0.17
SILKGraft-2 Plus	0.77 ± 0.10	5.07 ± 0.21
*t*-test ^1^	1 vs. 1 Plus and 2 vs. 2 Plus: *p* > 0.05 1/1 Plus vs. 2/2 Plus: *p* < 0.05 ^2^	

^1^*t*-test (confidence interval of the mean 95%). All data followed a normal distribution. *p* > 0.05: no statistical significance. *p* < 0.05: statistically significant difference. ^2^ All combinations were statistically analyzed.

**Table 3:** Thermal properties and degree of crystallinity.

SILKGraft Designs	DSC				FTIR-CI ^2^ (I_1260_/I_1230_)
	ES Peak Temp. (°C)	TEX Peak Temp. (°C)	ΔH (J/g)	w% ES	
SILKGraft-1	289 ± 2	318 ± 3	437 ± 43	60 ± 2	0.58 ± 0.01
SILKGraft-1 Plus	290 ± 1	318 ± 3	424 ± 40	63 ± 5	0.57 ± 0.02
SILKGraft-2	290 ± 1	320 ± 2	432 ± 36	51 ± 3	0.62 ± 0.01
SILKGraft-2 Plus	289 ± 1	321 ± 2	415 ± 42	52 ± 3	0.61 ± 0.02
*t*-test ^1^	Any combination: *p* > 0.05			1/1 Plus vs. 2/2 Plus: *p* < 0.05 ^3^	

^1^*t*-test (confidence interval of the mean 95%). All data followed a normal distribution. *p* > 0.05: no statistical significance. *p* < 0.05: statistically significant difference. ^2^ CI = crystallinity index. ^3^ All combinations were statistically analyzed.

**Table 4:** Mechanical properties: circumferential and uniaxial tensile tests.

SILKGraft designs	Circumferential Tensile Test		Uniaxial Tensile Test	
	Elastic Modulus (MPa)	Maximum Load (N)	Maximum Load (N)	Displacement at Maximum Load (mm)
SILKGraft-1	2.9 ± 0.4	30.2 ± 2.3	33.7 ± 2.5	38.2 ± 4.9
SILKGraft-1 Plus	2.9 ± 0.6	30.5 ± 3.2	30.0 ± 1.5	42.4 ± 8.1
SILKGraft-2	6.4 ± 1.7	83.9 ± 11.2	18.7 ± 2.8	206.4 ± 18.5
SILKGraft-2 Plus	5.1 ± 0.6	74.9 ± 10.4	20.2 ± 1.9	187.5 ± 26.5
*t*-test ^1^	1/1 Plus vs. 2/2 Plus: *p* < 0.05 ^2^			

^1^*t*-test (confidence interval of the mean 95%). All data followed a normal distribution. *p* > 0.05: no statistical significance. *p* < 0.05: statistically significant difference. ^2^ All combinations were statistically analyzed.

**Table 5:** Mechanical properties: pressurized burst strength, kink resistance, and compliance.

SILKGraft Designs	Pressurized Burst Strength		Kink Resistance (mm)	Compliance (ml/mmHg × 10^−3^)
	Burst Pressure Resistance (mmHg)	Longitudinal Damage Size (mm)		
SILKGraft-1	846 ± 207	1.48 ± 1.32	54 ± 0	3.23 ± 0.21
SILKGraft-2 Plus	1147 ± 126	0.62 ± 0.19	27 ± 6	3.75 ± 0.07
*t*-test ^1^	*p* > 0.05		Nd ^2^	*p* < 0.05

^1^*t*-test (confidence interval of the mean 95%). All data followed a normal distribution. *p* > 0.05: no statistical significance. *p* < 0.05: statistically significant difference. ^2^ Nd: not determined.

## Data Availability

The datasets generated for this study are available on reasonable request to the corresponding author.

## Supplementary Materials

The following supporting information can be downloaded at: https://www.mdpi.com/article/10.3390/ma15103735/s1, Figure S1: Set up of grips for mechanical testing: (A) circumferential tensile test. (B) Uniaxial tensile test.; Table S1: Values of centerline average roughness height (Ra), root mean square roughness height (Rq), and maximum peak to through height (Rt) measured in the longitudinal direction.; Table S2: Values of centerline average roughness height (Ra), root mean square roughness height (Rq), and maximum peak to through height (Rt) measured in the transversal direction.; Table S3: Average values of centerline average roughness height (Ra), root mean square roughness height (Rq), and maximum peak to through height (Rt) measured in the longitudinal direction; Table S4: Average values of centerline average roughness height (R_a_), root mean square roughness height (R_q_), and maximum peak to through height (R_t_) measured in the transversal direction.
