# Supplementary material for: Silk Vascular Grafts with Optimized Mechanical Properties for the Repair and Regeneration of Small Caliber Blood Vessels
Source: Materials (Basel). 2022 May 23;15(10):3735. doi: 10.3390/ma15103735 (PMC9147556; doi:10.3390/ma15103735)
Supplement: Supplementary file 1 [file materials-15-03735-s001.zip › materials-1722086-supplementary.pdf]

Supplementary Materials

Silk Vascular Grafts with Optimized Mechanical Properties for the Repair and Regeneration of Small Caliber Blood Vessels

Elisa Valsecchi [1], Marco Biagiotti [1], Antonio Alessandrino [1], Dario Gastaldi [2], Pasquale Vena [2] and Giuliano Freddi [1,*]

[1] Silk Biomaterials Srl, 22074 Lomazzo, Como, Italy; elisa@silkbiomaterials.com (E.V.); marco@silkbiomaterials.com (M.B.); antonio@silkbiomaterials.com (A.A.); giuliano@silkbiomaterials.com (G.F.)

[2] Department of Chemistry Materials and Chemical Engineering Giulio Natta; Laboratory of Biological Structures Mechanics-LaBS, Politecnico di Milano; dario.gastaldi@polimi.it (D.G.); pasquale.vena@polimi.it (P.V.)

* Correspondence: giuliano@silkbiomaterials.com

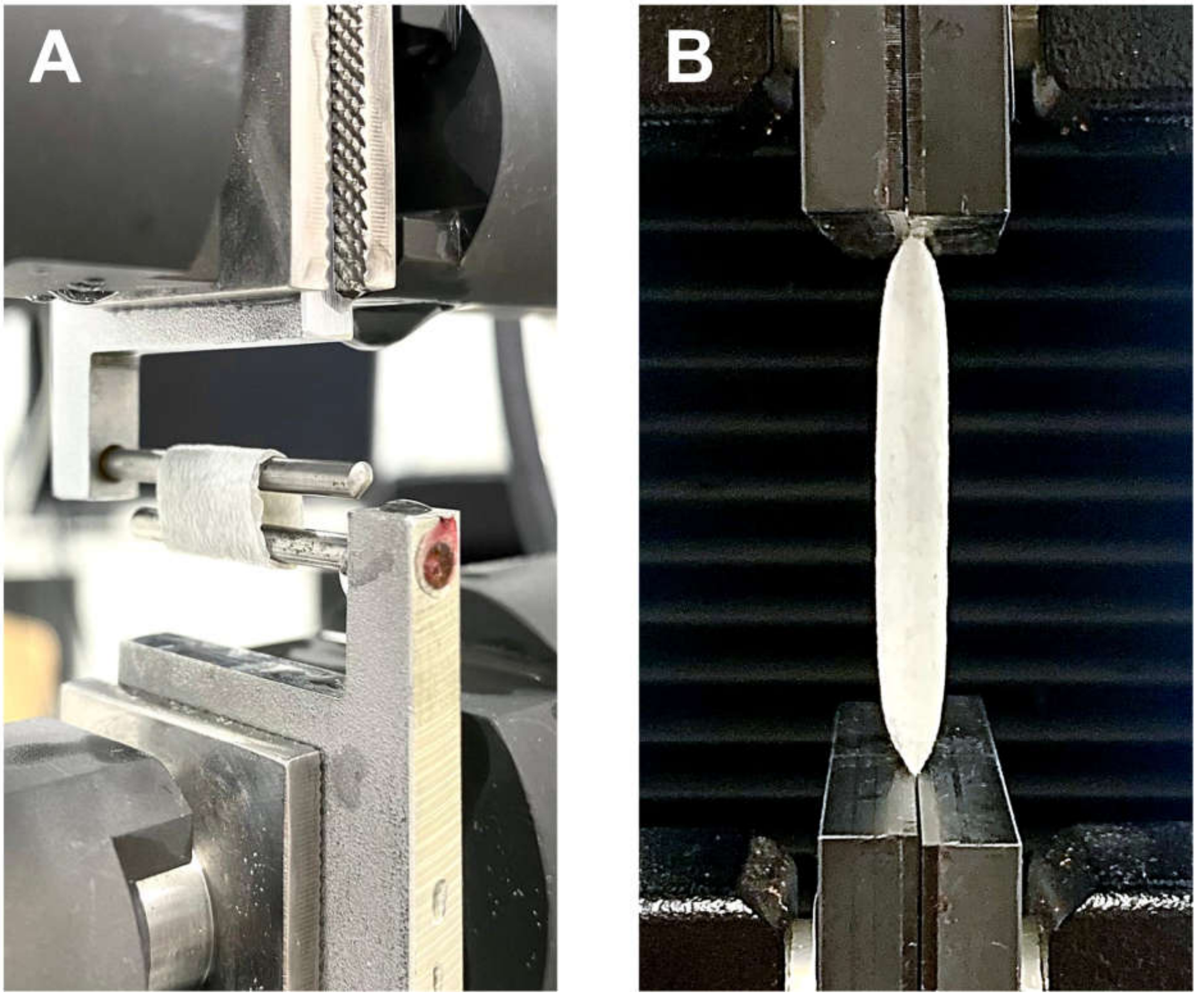

Figure S1. Set up of grips for mechanical testing: (A) Circumferential Tensile Test. (B) Uniaxial Tensile Test.

Table S1. Values of centerline average roughness height (R_a), root mean square roughness height (R_q), and maximum peak to through height (R_t) measured in the longitudinal direction.

Device variant	Sample	R_a (µm)	R_q (µm)	R_z (µm)
SILKGraft-1	1	13.2 ± 5.0	16.0 ± 6.3	60.0 ± 29.1
	2	14.7 ± 3.5	18.4 ± 4.4	61.0 ± 11.6
	3	21.8 ± 7.2	26.5 ± 7.5	83.6 ± 20.3
	4	13.3 ± 2.6	16.4 ± 3.0	50.9 ± 7.5
	5	17.3 ± 10.0	20.5 ± 11.1	68.0 ± 30.7
	6	9.7 ± 2.1	11.9 ± 2.6	40.2 ± 7.6
SILKGraft-2	1	34.5 ± 5.8	42.7 ± 7.4	160.8 ± 22.4
	2	20.0 ± 5.9	25.1 ± 6.4	96.6 ± 19.1
	3	31.7 ± 4.3	39.8 ± 5.0	151.9 ± 17.8
	4	33.8 ± 3.6	41.5 ± 4.9	157.8 ± 12.3
	5	34.7 ± 3.4	43.4 ± 3.8	153.4 ± 18.0
	6	25.1 ± 2.8	30.5 ± 3.6	108.6 ± 13.1

Table S2. Values of centerline average roughness height (R_a), root mean square roughness height (R_q), and maximum peak to through height (R_t) measured in the transversal direction.

Device variant	Sample	R_a (µm)	R_q (µm)	R_z (µm)
SILKGraft-1	1	18.0 ± 2.4	23.3 ± 2.1	75.8 ± 11.5
	2	15.9 ± 1.3	20.9 ± 1.9	64.9 ± 8.1
	3	21.3 ± 6.3	26.5 ± 8.6	90.5 ± 25.7
	4	22.4 ± 1.9	28.4 ± 1.6	93.3 ± 4.2
	5	29.7 ± 6.4	36.0 ± 6.8	112.1 ± 19.2
	6	17.9 ± 2.5	24.1 ± 2.8	85.5 ± 13.9
SILKGraft-2	1	21.6 ± 4.3	26.3 ± 4.8	88.6 ± 10.4
	2	22.3 ± 2.1	28.6 ± 3.2	100.8 ± 13.6
	3	29.1 ± 3.1	35.6 ± 4.0	103.4 ± 8.6
	4	26.6 ± 5.6	33.0 ± 5.6	101.5 ± 8.7
	5	26.0 ± 3.4	32.0 ± 2.9	109.6 ± 6.1
	6	19.6 ± 2.1	24.6 ± 2.8	84.9 ± 4.8

Table S3. Average values of centerline average roughness height (R_a), root mean square roughness height (R_q), and maximum peak to through height (R_t) measured in the longitudinal direction.

Device variant	R_a (μm)	R_q (μm)	R_z (μm)
SILKGraft-1	15.0 ± 6.5	18.3 ± 7.5	60.6 ± 22.9
SILKGraft-2	30.0 ± 6.9	37.2 ± 8.5	138.2 ± 30.5

Table S4. Average values of centerline average roughness height (R_a), root mean square roughness height (R_q), and maximum peak to through height (R_t) measured in the transversal direction.

Device variant	R_a (μm)	R_q (μm)	R_z (μm)
SILKGraft-1	20.9 ± 5.9	26.5 ± 6.6	87.0 ± 20.6
SILKGraft-2	24.2 ± 4.7	30.0 ± 5.3	98.1 ± 12.1
